# Obituaries

**Published:** 1861-01

**Authors:** A. Berry


### OBITUARIES.

BY A. BERRY.

It is usual to see in the newspapers obituary notices in which the writers draw largely on their imaginations. I think that in general such notices of dentists are truthfully written.

It may seem out of place to criticise such, and especially in connection with the death of the lamented Professor Harris. But in his obituary in the October number of the New York *Dental Journal*, is, I think, an error which, had it occurred in Dr. H.'s lifetime, he would have corrected at once. It is stated that "in 1840 he (Dr. H.) founded the Baltimore College of Dental Surgery." If I am not mistaken Dr. Hayden acted no unimportant part in connection with Dr. H. in founding this school.

There is another error, as it seems to me, in the preamble to the resolutions passed at the meeting of dentists lately in New York, which asserts of the Baltimore College of Dental Surgery, that it was "for a long period the only Dental College in the world." Was not the Ohio College of Dental

Surgery founded five years after that at Baltimore? Is that truly a "*long period?*" Would any one unacquainted with the facts suppose, from reading this preamble, that only five short years were intended to be understood by this "long period?"

